# Yeast adaptive response to acetic acid stress involves structural alterations and increased stiffness of the cell wall

**DOI:** 10.1038/s41598-021-92069-3

**Published:** 2021-06-16

**Authors:** Ricardo A. Ribeiro, Miguel V. Vitorino, Cláudia P. Godinho, Nuno Bourbon-Melo, Tiago T. Robalo, Fábio Fernandes, Mário S. Rodrigues, Isabel Sá-Correia

**Affiliations:** 1 iBB–Institute for Bioengineering and Biosciences, Instituto Superior Técnico, Universidade de Lisboa, Av. Rovisco Pais, 1049-001 Lisbon, Portugal; 2 Associate Laboratory i4HB—Institute for Health and Bioeconomy at Instituto Superior Técnico, Universidade de Lisboa, Av. Rovisco Pais, 1049-001 Lisbon, Portugal; 3 Department of Bioengineering, Instituto Superior Técnico, Universidade de Lisboa, Av. Rovisco Pais, 1049-001 Lisbon, Portugal; 4 BioISI–Biosystems and Integrative Sciences Institute, Faculdade de Ciências, Universidade de Lisboa, 1749-016 Lisbon, Portugal; 5 Departament of Physics, Faculdade de Ciências, Universidade de Lisboa, 1749-016 Lisbon, Portugal

**Keywords:** Fungal physiology, Applied microbiology

## Abstract

This work describes a coordinate and comprehensive view on the time course of the alterations occurring at the level of the cell wall during adaptation of a yeast cell population to sudden exposure to a sub-lethal stress induced by acetic acid. Acetic acid is a major inhibitory compound in industrial bioprocesses and a widely used preservative in foods and beverages. Results indicate that yeast cell wall resistance to lyticase activity increases during acetic acid-induced growth latency, corresponding to yeast population adaptation to sudden exposure to this stress. This response correlates with: (i) increased cell stiffness, assessed by atomic force microscopy (AFM); (ii) increased content of cell wall β-glucans, assessed by fluorescence microscopy, and (iii) slight increase of the transcription level of the *GAS1* gene encoding a β-1,3-glucanosyltransferase that leads to elongation of (1→3)-β-d-glucan chains. Collectively, results reinforce the notion that the adaptive yeast response to acetic acid stress involves a coordinate alteration of the cell wall at the biophysical and molecular levels. These alterations guarantee a robust adaptive response essential to limit the futile cycle associated to the re-entry of the toxic acid form after the active expulsion of acetate from the cell interior.

## Introduction

The mechanistic understanding of yeast adaptation and tolerance to environmental stresses is not only a highly challenging and relevant topic in biological research but is essential for guiding the construction of superior industrial strains or for the efficient control of the deleterious activity of spoilage yeasts^1^. Therefore, the elucidation of the mechanisms underlying adaptation and tolerance to acetic acid in yeasts is of high relevance in biotechnology and food industry^1,2^. In fact, acetic acid is (i) a major inhibitory compound present in lignocellulosic hydrolysates affecting their use in sustainable biorefinery processes; (ii) produced during normal yeast metabolism in biotechnological processes contributing to growth and fermentation inhibition or even arrest, and (iii) a widely used preservative in foods and beverages^2,3^.

At a pH below acetic acid p*K*_a_ (4.75 at 25 °C)^4^, acetic acid is able to passively diffuse through the plasma membrane lipid bilayer. Once inside the cell, at the near-neutral cytosol, acetic acid dissociates leading to the release of protons (H^+^), causing the decrease of intracellular pH (pHi), and the accumulation of the acetate counter-ion (CH_3_COO^−^), the inhibition of metabolism, oxidative stress and increased turgor pressure^1,2^. In recent years, several chemogenomic^5,6^, transcriptomic^5,7–12^ and proteomic^13,14^ studies allowed a more comprehensive understanding of the global mechanisms involved in *Saccharomyces cerevisiae* response and tolerance to acetic acid. Among them is the alteration of the molecular composition and physical properties of plasma membrane and cell wall, leading to the decrease of cell envelope permeability^2,15–17^. Such adaptation, at the level of the cell envelope, is essential to reduce the diffusion rate of this weak acid from the cell exterior to the intracellular medium. This response counteracts the re-entry of the acid form after the active expulsion of acetate from the cell interior, presumably catalysed by efflux pumps (e.g. Tpo2, Tpo3, Aqr1, Pdr18)^18^ and, in this way, limits the associated futile cycle^2,19^.

Several genes encoding proteins required for the synthesis of cell wall polysaccharides and cell wall remodeling are transcriptionally responsive to acetic acid stress and/or determinants of acetic acid stress tolerance^5–7,9,11^. The *YGP1* and *SPI1* genes, encoding a cell wall-glycoprotein and a Glycosylphosphatidylinositol (GPI)-anchored cell wall protein, respectively, are determinants of acetic acid tolerance and directly up-regulated in response to acetic acid by Haa1, the major regulator in adaptive response and tolerance to acetic acid in *S. cerevisiae*^5–7,9^. Genes involved in 1-3 β-glucan synthesis (*FKS1, ROM2),* 1-6 β-glucan synthesis (*KRE6),* 1-3 β-glucan elongation and branching (*GAS1*), chitin synthesis (*CHS1, CHS5*) and cell wall protein mannosylation (*MNN2, MNN9, MNN11, KTR4)* are also reported determinants of tolerance to acetic acid^6^. Although this seems to point towards a role for the cell wall in acetic acid stress response and tolerance, the transcript levels from the acetic acid tolerance determinant genes *CHS1*, *KTR4*, *MNN9* and *FKS1* were reported to decrease in acetic acid stressed cells^7^. Another transcriptomic analysis reports the differential-expression of 28 cell wall metabolism-related genes under acetic acid stress from which 24 were down-regulated, in particular *KRE6*, *FKS1* and its paralog *FKS2*/*GSC2*^11^.

The mechanisms involving changes in the chemical structure and organization of the cell wall impact its biophysical properties^20^. For instance, the stiffness of the cell wall appears to be strongly dependent on the molecular architecture of the cell wall, particularly on the cross-linking between β-glucans and chitin, rather than on the increase of a particular cell wall polysaccharide or on cell wall thickness^21,22^. Although nanomechanical and biochemical changes occurring at the yeast cell wall in response to heat and ethanol stresses have been reported^23–25^, the response of the cell wall to stress induced by acetic acid is so far unknown and on the focus of this study. The objective of the present work was to examine the hypothesized involvement of the remodeling of the cell wall at the molecular and biophysical levels during the time course of the adaptive response of *S. cerevisiae* to a sub-lethal concentration of acetic acid. The alterations occurring at the level of yeast cell wall architecture during adaptation were assessed based on cell wall susceptibility to lyticase activity and on cell wall stiffness assessed by atomic force microscopy (AFM). The transcriptional activation of genes involved in cell wall synthesis and remodeling was assessed by qRT-PCR and the content of cell wall polysaccharides by fluorescence microscopy. Results provide a global view on mechanisms underlying the time-course of yeast cell adaptation to acetic acid stress at the cell wall level.

## Results

### Yeast adaptation to acetic acid involves increased cell wall resistance to lyticase activity

**Figure 1:**
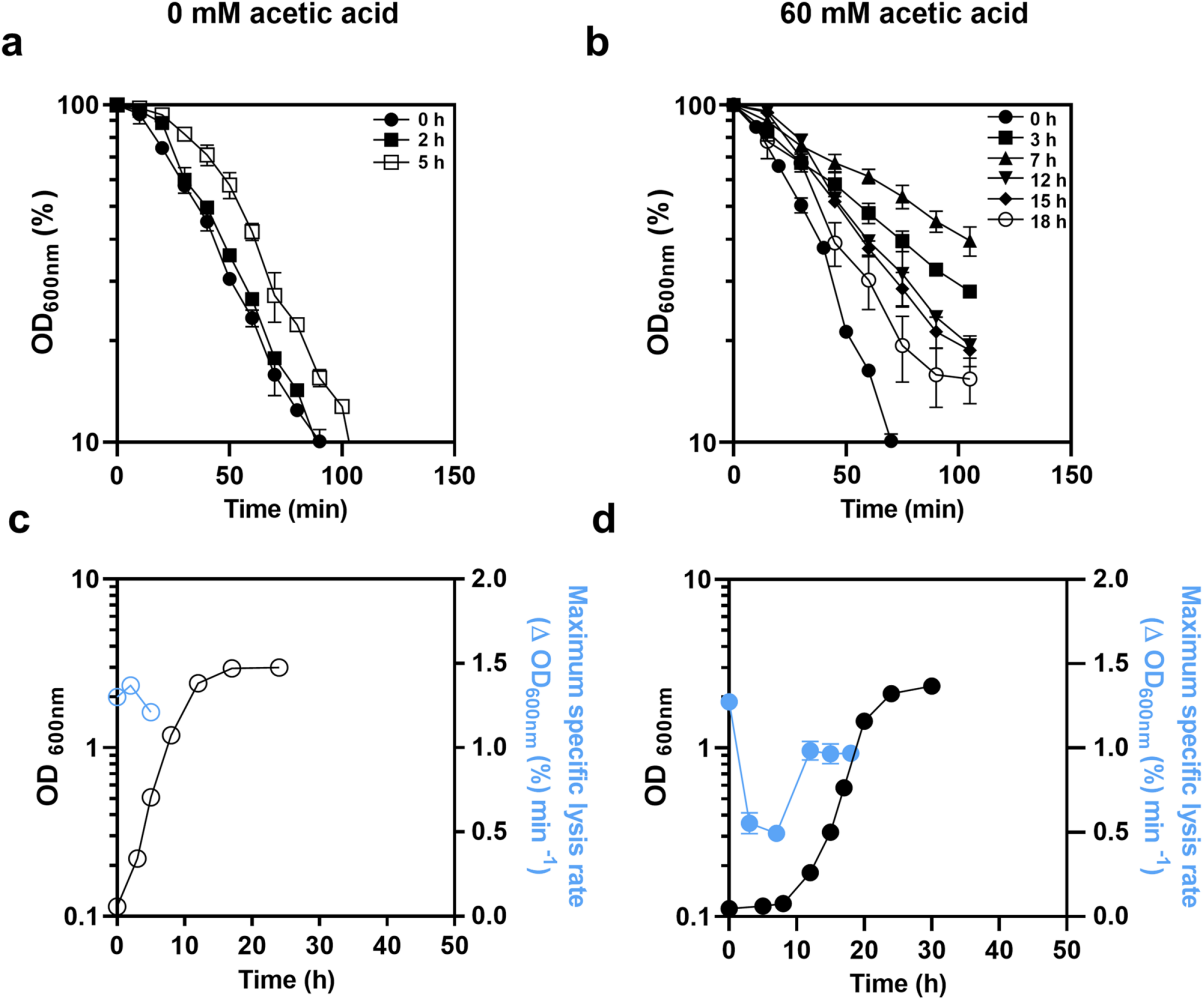
Effect of lyticase in yeast cell wall during the growth curve in the absence or presence of acetic acid stress. Decrease of the OD_600nm_ of *S. cerevisiae* BY4741 cell suspensions (in %) following the addition of lyticase, as described in M&M. Cells were harvested at 0 h (filled circle), 2 h (filled square), 5 h (open square) of cultivation in MM4 pH 4.0 not supplemented with acetic acid (**a**) and at 0 h (filled circle), 3 h (filled square), 7 h (filled triangle), 12 h (filled down pointing triangle), 15 h (filled rhombus) and 18 h (open circle) of cultivation in the presence of 60 mM acetic acid at 4.0 (**b**).The maximum specific lysis rates, determined based on data in (**a**) and (**b**) for yeast cells harvested at the selected time-points, are shown during cultivation in the absence (**c**) or presence of acetic acid (**d**). The initial biomass concentration used corresponded to culture OD_600nm_ of 0.1 ± 0.01. Data are means from at least three independent experiments and bars represent standard deviation.

The alterations occurring in yeast cell wall architecture during cultivation in MM4 pH 4.0, either supplemented or not with 60 mM acetic acid, were monitored based on cell wall susceptibility to lyticase activity. This technique was previously shown to be valuable to monitor alterations in the cell wall in response to various environmental stresses^26–28^. The maximum specific lysis rate for a given timepoint was calculated as the slope of the straight line that best fits the semi-logarithmic plot of the time-course decrease of cell suspension OD_600nm_ after the addition of lyticase (Fig. 1a,b). Values were plotted during the growth curve (Fig. 1c,d). The maximum specific lysis rate exhibits similar values during exponential growth (0–5 h) in the absence of acetic acid (Fig. 1a,c). However, for cells cultivated in medium supplemented with 60 mM acetic acid (pH 4.0), a rapid and marked significant reduction of the maximum specific lysis rate occurs at 3 h of acetic acid-induced latency, compared to the initial time-point (from 1.27 to 0.55 Δ[OD_600nm_(%)] min^−1^; *p* < 0.00001; one-way-ANOVA) (Fig. 1b,d). The lowest maximum specific lysis rate was attained after 7 h of growth latency (Fig. 1b,d). Moreover, acetic acid-adapted exponentially-growing cells exhibit a significantly lower susceptibility to lyticase compared with exponentially-growing unstressed cells (*p* = 0.002; one-way ANOVA; Fig. 1c,d). These results indicate that adaptation to acetic acid leads to significant cell wall architecture alterations, resulting in higher resistance to lyticase activity.

### Yeast cell adaptation to acetic acid stress involves the increase of yeast cell wall stiffness

**Figure 2:**
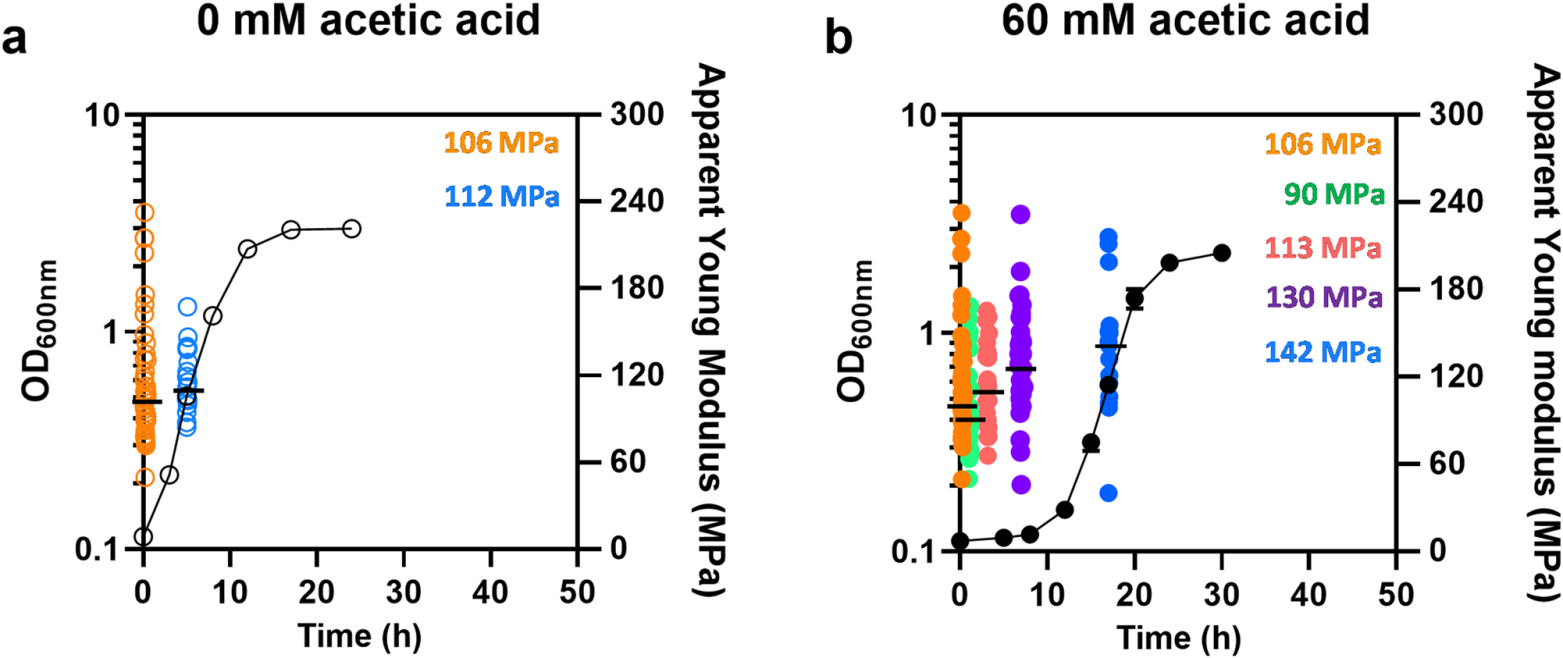
Apparent Young’s modulus of yeast cells during the growth curve in the absence or presence of acetic acid stress. Time-course analysis of the yeast cell surface stiffness, assessed by atomic force microscopy in BY4741 cells cultivated in the absence (**a**) or presence of acetic acid (**b**), as described for Fig. 1. The stiffness of the cell surface is represented as the apparent Young’s modulus. Each point in the graph corresponds to the median value of about 100 curves over a single cell. For each condition, at least, 15 cells were analyzed from, at least, 3 independent experiments.

The biophysical properties of the yeast cell surface during adaptation to acetic acid stress were examined by AFM to assess the Young’s modulus, which is the quantitative expression of the cell surface elasticity that reflects cell wall stiffness. The Young’s modulus was not found to vary significantly during the first 5 h of exponential growth in MM4 pH 4.0 (Fig. 2a). Exposure to 60 mM of acetic acid (pH 4.0) was found to lead to the increase of the Young’s modulus value during the induced growth latency; after 7 h of incubation, the cell population exhibits a significantly higher Young’s modulus than unstressed cells (Fig. 2; *p* = 0.006; one-tailed Mann–Whitney U test). The Young’s modulus of exponentially-growing adapted cells in the presence of acetic acid (142 MPa) is also significantly higher than the Young’s modulus of the corresponding unstressed cells (Fig. 2; *p* = 0.002; one-tailed Mann–Whitney U test). These results indicate that adaptation to acetic acid-induced stress involves the increase in yeast cell wall stiffness.

### Transcriptional profiles of cell wall biosynthesis-related genes in response to acetic acid

**Figure 3:**
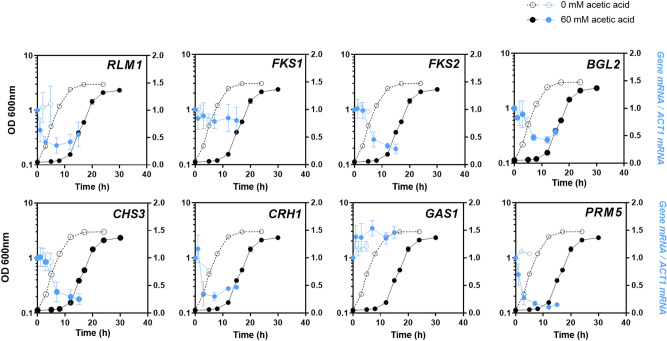
Levels of mRNA from cell wall biosynthesis-related genes during yeast cultivation in the absence or presence of acetic acid. Time-course of the mRNA levels from *RLM1*, *FKS1*, *FKS2*, *BGL2, CHS3*, *CRH1, GAS1* and *PRM5* genes during cultivation of BY4741 in absence (open circle) or presence (filled circle) of acetic acid, as described for Fig. 1. The transcriptional levels (in blue) from the indicated genes were assessed by qRT-PCR, using *ACT1* as the internal control. The relative value obtained for each target gene at the initial time-point under unstressed conditions was set as 1. Results are means of, at least, three biological replicates and error bars represent standard deviation. Primers were designed using Primer Express V3.0 (Applied Biosystems).

Quantitative real-time Reverse Transcription–PCR (qRT-PCR) was performed to assess the levels of transcription from several cell wall biosynthesis-related genes during cultivation in the absence or presence of 60 mM acetic acid, at pH 4.0. The chosen genes were: *RLM1* (encoding a transcription factor responsible for the transcriptional activation of the majority of genes induced in response to cell wall stress through the CWI pathway^29^), *FKS1* and *FKS2* (encoding β-1-3-glucan synthases^30^), *BGL2* (encoding endo-beta-1,3-glucanase^31^), *CHS3* (encoding a major chitin synthase^32^), *CRH1* (encoding a chitin transglycosylase^33^), *GAS1* (encoding a β-1,3-glucanosyltransferase, an important enzyme for cell wall remodeling involving elongation of (1→3)-β-d-glucan chains and branching^31,34^), and *PRM5* (a Rlm1 target and the hallmark of CWI pathway activation^29^). The mRNA levels from these genes were not found to vary significantly during the first 5 h of exponential growth in MM4, at pH 4.0 (Fig. 3). However, when the medium was supplemented with acetic acid, the mRNA levels from all genes tested, except for *GAS1* and *FKS1,* were found to decrease markedly during growth latency. The mRNA levels from *FKS1* were similar during cultivation in the presence of acetic acid but the transcript levels from *GAS1* moderately increased to around 1.5-fold higher than the levels of expression in unstressed cells, throughout the time-points tested (Fig. 3). The maximum activation was obtained after 7 h of cultivation in the presence of acetic acid (1.54-fold the levels of exponentially-growing unstressed cells; *p* = 0.001421; one-way ANOVA).

Collectively, our results show that cultivation in the presence of acetic acid leads to the down-regulation of transcription from several cell wall biosynthesis-related genes, while the β-1,3-glucanosyltransferase encoding gene *GAS1* was up-regulated.

### Yeast cell adaptation to acetic acid stress involves the increase of cell wall β-glucans, as assessed by fluorescence microscopy

**Figure 4:**
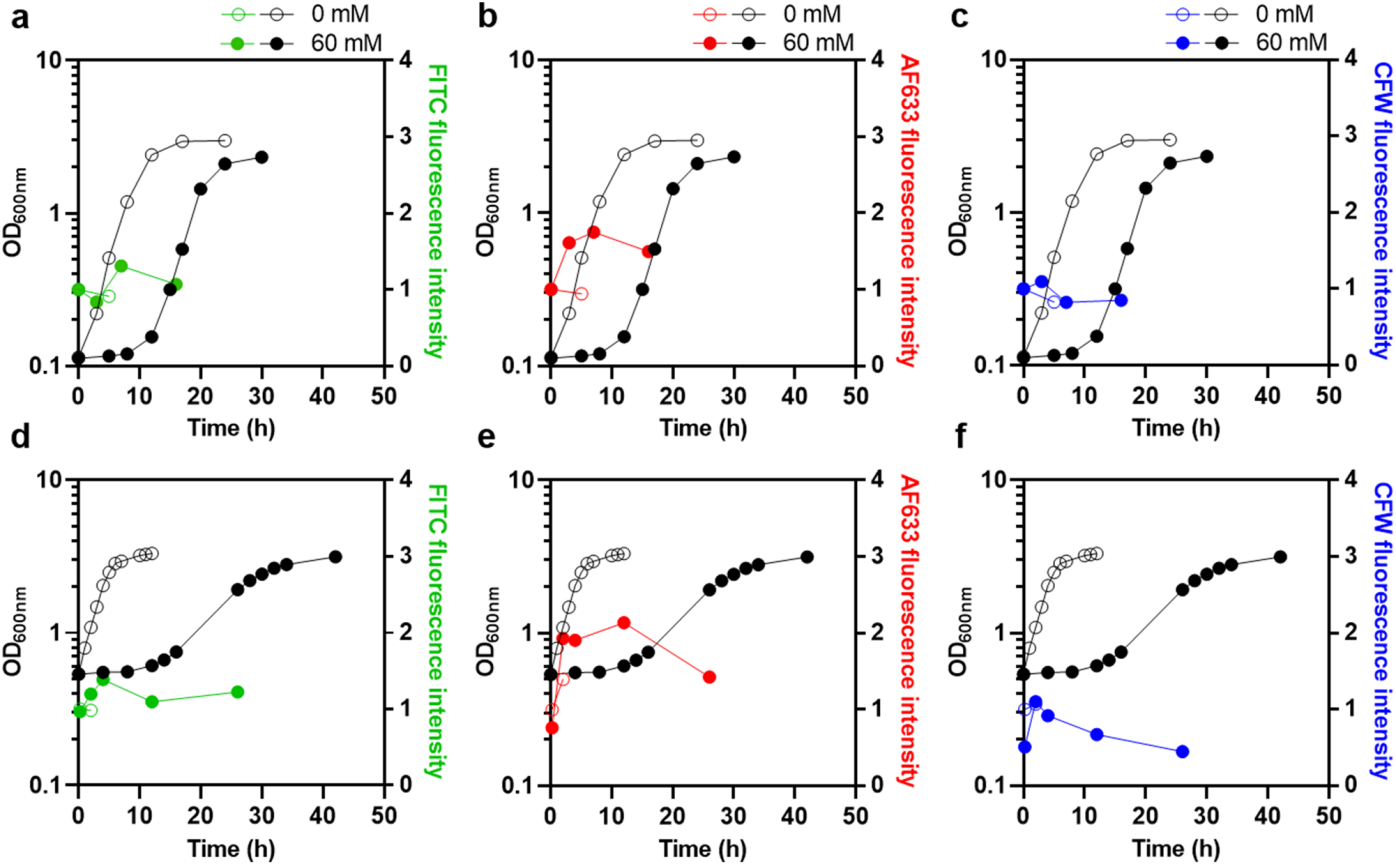
Content of cell wall polysaccharides in yeast cells during adaptation to acetic acid stress by fluorescence microscopy. Quantitative analysis of the cell wall polysaccharides during cultivation of *S. cerevisiae* BY4741 in the absence (open circle) or presence (filled circle) of acetic acid, as described for Figs. 1, 2, 3 (**a**–**c**). Results of an independent experiment using a higher level of acetic acid stress and a higher initial biomass concentration (corresponding to initial OD_600nm_ of 0.5 ± 0.05) are also shown (**d**–**f**). The cell wall components mannans (green), β-glucans (red) and chitin (blue) were stained with Concanavalin A conjugated with Fluorescein (FITC), Fc-Dectin 1 conjugated with Alexa Fluor 633 (AF633) and Calcofluor White (CFW), respectively. Quantification of the fluorescence intensity was performed with a Leica TCS SP5 (Leica Microsystems CMS GmbH, Manheim, Germany) inverted confocal microscope (DMI600). Median fluorescence intensity values result from the analysis of, at least, 34 cells obtained from two replicates. Individual cell measurements can be consulted in Supplementary Figure S2.

Possible changes in the polysaccharide content of cell wall during cultivation in the presence of an identical acetic acid stress were assessed based on their quantitative analysis by fluorescence microscopy. Images illustrating those obtained by fluorescence microscopy are shown in Supplementary Figure S1. To make it easier to visualize and analyze the results, Fig. 4 shows only the median values obtained for cells harvested during stressed and unstressed cultivations. Individual cell measurements can be consulted in Supplementary Figure S2.

Results show that in growth medium supplemented with 60 mM acetic acid (pH 4.0), the cell wall content in β-glucans increased, as quantified by the fluorescence intensity of Alexa Fluor 633 conjugated anti-human IgG with FC-Dectin1 (Fig. 4b). Yeast exposure to acetic acid leads to a significant increase in fluorescence intensity at 3 h of cultivation (1.6-fold higher than the level of the exponentially-growing unstressed cells; *p* < 0.00001; one-tailed Mann–Whitney U test) (Fig. 4b). The levels of β-glucans attained were maintained significantly higher than those in unstressed cells (*p* < 0.00001; one-tailed Mann–Whitney U test) even when acetic acid-grown cells reached exponential phase of growth (Fig. 4b). The increase of the glucan content in yeast cells adapting and adapted to acetic acid was confirmed using a higher level of stress (Fig. 4e).

Concerning the content in mannans and chitin, quantified by fluorescence intensity of Concanavalin A-fluorescein (FITC) and Calcofluor White (CFW), respectively, its consistent alteration during the whole period of cultivation in the presence of acetic acid, when compared with unstressed cultivation, was not so clear for the two tested conditions (Fig. 4a,c,d,f). However, the mannan content of yeast cells adapting or growing in conditions leading to higher acetic acid stress was found to be significantly higher compared with unstressed cells (Figs. 4d and S2d). For the chitin content, considering the time-course profile obtained and the fact that calcofluor white can also stain some beta-glucans, possibly interfering with chitin quantification, no definitive conclusions could be taken.

## Discussion

Among the mechanisms that have been proposed to underlie *Saccharomyces cerevisiae* adaptation and tolerance to acetic acid is the remodeling of the cell envelope^2^. They are considered essential to limit the diffusional re-entry of the acid form of the weak acid following the induced active expulsion of acetate from the cell interior, thus counteracting this futile cycle^2,19,35^. The present work describes, for the first time, a coordinated and comprehensive view on the time-course of the alterations occurring at the level of the cell wall in a yeast cell population during adaptation to sudden exposure to a sub-lethal stress induced by acetic acid. The coordinated alteration of cell wall susceptibility to the action of lyticase activity, cell wall polysaccharide composition and nanomechanical properties, and of the transcription levels of key cell wall-biosynthesis-related genes, following yeast exposure to acetic acid was systematically examined. Collectively, results reinforce the notion that the adaptive yeast response to acetic acid stress involves a coordinate alteration of the cell wall at the biophysical and molecular levels.

The increased resistance to lyticase observed in this study for yeast cells during adaptation to acetic acid was reported before by our laboratory for other weak acids^28^. The marked increase in cell wall resistance to lyticase (containing β-(1→3)-glucan laminaripentaohydrolase with additional β-(1→3)-glucanase, protease, and mannanase activities), during the first hours of exposure to acetic acid strongly suggests that changes occurred in the cell wall composition and/or cross-linking between cell wall macromolecules. Mechanisms involving the synthesis and reorganization of cell wall constituents have been proposed to be relevant to maintain cell wall integrity and to decrease cell permeability to weak acids and thus to limit their contact with plasma membrane, therefore reducing membrane damage and intracellular acidification^2,5,16,28,35–37^. In fact, it was demonstrated that pre-exposure to acetic acid or sorbic acid causes a decrease in the rate of intracellular acidification, most likely as a result of changes occurring in plasma membrane and cell wall^19^. The mechanism to reduce intracellular acidification based on the active extrusion of protons and of the acid counter-ion is not, by itself, efficient unless the cell is able to restrict the diffusional re-entry of the weak acid^2,19,35^*.* Plasma membrane permeability was found to increase upon exposure to acetic acid stress and to decrease following yeast cell adaptation to the stress and growth resumption in its presence, although maintaining permeability levels above those in the corresponding unstressed cells^15^. Remarkably, the minimum value of cell susceptibility to lyticase was found during the latency phase, while the susceptibility to lyticase of cells exponentially growing in the presence of the acid was higher but below the values for unstressed cells. Results are consistent with the concept that cell wall remodeling is crucial during acetic acid-induced latency when the permeabilization of stressed yeast cells is maximal, thus limiting the deleterious effects of the acid.

The present work provides, for the first time, a time course characterization of the nanomechanical properties of the cell wall by assessing cell surface stiffness of the yeast population following sudden exposure to stress during the full length of the cultivation in medium supplemented with a sub-lethal concentration of acetic acid stress. Due to the plastic nature of the cell wall, stress-induced mechanisms that include changes in composition and/or remodeling of the cell wall may result in changes of its biophysical properties^20^. According to the data of the present study, cell surface stiffness increases during adaptation to acetic acid stress. The stiffness of the cell wall is apparently not linked to changes in a specific component of the cell wall, but rather related with its molecular architecture, involving the cross-link between cell wall components, in particular the cross-link between β-glucans and chitin that seem to have a prominent role in cell wall stiffness^21,24^. The observed correlation between the time-course profiles of the increase of *GAS1* transcription level and the content of glucan in the cell wall suggests that cell wall remodeling under acetic acid stress is, at least partially, due to the β-1,3-glucanosyltransferase activity of Gas1 involved in glucan elongation and also in branching^31,34^, while no support for the de novo synthesis of more glucan was obtained. Consistent with these results, *GAS1* is a demonstrated determinant of acetic acid tolerance^6^ as well as for other environmental stresses such as those induced by H_2_O_2_ treatment^38^, low pH^39^ and ethanol^40^. On the contrary, the transcription levels from *FKS2*, *BGL2*, *CHS3*, *CRH1 and PRM5* genes suffer a continuous reduction during the whole period of stressed cultivation. The mRNA levels from *RLM1*, encoding the major transcriptional regulator of the CWI pathway effectors, were also reduced by cultivation in the presence of this weak acid, consistent with the down-regulation of Rlm1-target genes observed herein. Consistent with previous transcriptomic analysis of cells exposed to sub-lethal and severe acetic acid stress, the observed down-regulation of *RLM1* also suggests that the CWI pathway may not be the major key player in acetic acid stress response^7,11^. However, since the CWI pathway gene *GAS1* was found to be upregulated under acetic acid stress, it is not possible, at this time, to put aside the possible role of this pathway in the adaptive response. Moreover, the expression of *FKS1* does not exhibit the same down-regulation pattern under acetic acid stress of other CWI pathway genes, *FKS1* mRNA levels being similar to those in unstressed cells. The maintenance of at least one of the glucan synthases encoded by the paralogue genes *FKS1* and *FKS2* in acetic acid stressed cells was found to be required^37^. Although other enzymes, responsible for glucan remodeling and/or glucan/chitin cross-linking or other alterations of cell wall structural composition may also be involved in cell wall remodeling under acetic acid stress, no evidence for this was obtained here.

The present work examined the involvement of the cell wall, at the molecular and biophysical levels, in *S. cerevisiae* adaptation to a sub-lethal concentration of acetic acid. Collectively, our results suggest that this adaptation involves changes in yeast cell wall composition and structure that result in a stiffer and more robust cell wall essential to limit the futile cycle associated to the re-entry of the toxic acid form, after the active expulsion of acetate from the cell interior.

## Methods

### Yeast strains and growth conditions

*The S. cerevisiae* parental strain BY4741 (*MATa, his3Δ1, leu2Δ10, met15Δ0, ura3Δ0*), obtained from the EUROSCARF collection, was used in this study. The strain was maintained at − 80 °C in YPD media supplemented with 30% (v/v) glycerol. Cells were cultivated at 30 °C, with orbital agitation (250 rpm), in minimal growth medium supplemented with amino acids and uracil (MM4). MM4 contains 1.7 g/L yeast nitrogen base without amino acids and ammonium sulphate (Difco, Michigan, USA), 20 g/L glucose (Merck, Darmstadt, Germany), 2.65 g/L (NH_4_)_2_SO_4_ (Panreac AppliChem, Connecticut, USA), 20 mg/L methionine, 20 mg/L histidine, 60 mg/L leucine and 20 mg/L uracil (all from Sigma, Missouri, USA). The medium pH was adjusted to 4.0 with HCl. For the majority of the experiments performed under acetic acid stress, MM4 medium was supplemented with 60 mM acetic acid (Fluka, Waltham, USA) using a solution of 5 M acetic acid, set to pH 4.0 with NaOH. For the majority of the growth experiments, MM4 was inoculated to an initial optical density at 600 nm (OD_600nm_) of 0.1 ± 0.05 mid-exponential cells harvested by filtration (Whatman, Maidstone, UK) from cultivation in fresh MM4 medium (pH 4.0) without acetic acid supplementation. Growth was followed by measuring culture OD_600nm_.

### Yeast cell wall susceptibility to lyticase

To monitor yeast cell wall structural alterations during cultivation in MM4 supplemented or not with 60 mM acetic acid, a lyticase susceptibility assay (β-1,3-glucanase from *Arthrobacter luteus*; Sigma (batch SLBS4644), containing β-(1→3)-glucan laminaripentaohydrolase with additional β-(1→3)-glucanase, protease, and mannanase activities) was conducted as described before^28^. Briefly, the parental strain was cultivated in MM4 liquid medium, either supplemented or not with 60 mM acetic acid, at pH 4.0, and were harvested by filtration at adequate time-points of the cultivation. Cells were washed with distilled water and used to inoculate 100 mL erlenmeyer flasks containing 50 mL 0.1 M phosphate buffer (pH 6.6), to a final OD_600nm_ of 0.5. After the addition of 15,000 U/mL of lyticase, cell lysis was followed by measuring the decrease of OD_600nm_ for each cell suspension and converting it to a percentage of the initial OD_600nm_ value. The susceptibility to lyticase is represented as the maximum specific lysis rate defined as the absolute value of the the slope of the straight line that best fits the semi-logarithmic plot of the linear part of the lysis curve.

### Assessment of the nanomechanical properties of yeast cell wall by atomic force microscopy (AFM)

For AFM assessment of cell wall nanomechanical properties, cells of the parental strain were grown and harvested at adequate time-points as described for lyticase susceptibility assays. Cell suspensions of OD_600nm_ of 0.2 were prepared in 5 mL of bi-distillate de-ionized water. These cell suspensions were filtered through a polycarbonate membrane (Whatman) with a pore size of 3 μm, similar to the yeast cell longitudinal size, and washed once with 15 mL bi-distillate de-ionized water. The filter was allowed to dry on air and then deposited on a piece of double-faced tape and placed on the AFM stage for analysis.

For imaging and Force-Distance measurement, the samples were analysed with a PicoLE Molecular Imaging system operated in contact mode. A microlever MSCT-F (Bruker, Billerica, USA) with nominal stiffness of 0.6 N/m and nominal tip radius of 10 nm was used in all experiments.

Force spectroscopy mapping, consisting of 32 × 32 approach/retract force–displacement curves was performed in the yeast cell surface. The maximum deflection of the AFM cantilever was set constant yielding a maximum applied force of ≈ 30 nN. The tip-sample approach velocity was about 0.5 μm/s. To this end, an area in the cell was selected so that no bud scars were included, resulting in a total of about 100 approach/retract force–displacement curves from which the median value was kept. The force distance grids were analysed for determination of the apparent Young’s modulus which is the quantitative expression of the cell surface elasticity that reflects the stiffness. The Young’s modulus was obtained by adjusting the DMT contact model to the approach curves. The force distance grids were processed by home-written software using Wolfram Mathematica (Wolfram Research, Illinois, USA). A total of 32 cantilevers were used and the spring constant of the cantilevers was calibrated using the Sader method^41^. In order to reduce bias due to different cantilevers being used on different cells, each cantilever was used to measured cells at several time-points investigated, and the process was randomized. Eventually, however, it was not possible to use the same cantilever for all conditions tested. Results arise from, at least, 3 independent experiments and from the analysis of, at least, 15 cells in each condition.

### Transcriptional analysis of cell wall biosynthetic and regulatory genes

For gene transcription assays, parental strain cells were harvested during cultivation as described above for AFM and lyticase assays. Total RNA extraction was performed by the hot phenol method^42^.

The quantitative real-time Reverse Transcription–PCR (qRT-PCR) protocol used to determine the mRNA levels from *RLM1*, *FKS1*, *FKS2*, *BGL2*, *CHS3*, *CRH1*, *GAS1* and *PRM5* genes followed the manufacturer’s instructions. Primer Express software V3.0 (Applied Biosystems) was used for primer design for the amplification of each target cDNA (Supplementary Table S1). The RT–PCR reaction was conducted in a thermal cycler block (Cleaver GTC965) and the qPCR was conducted in QuantStudio 5 (Applied Biosystems) using NZYSpeedy qPCR Green Master Mix (NZYTech). The *ACT1* mRNA level was used as the internal control (Supplementary Table S1). The relative value obtained for each target gene at the initial time-point at 0 h under unstressed conditions was set as 1 and the remaining values are relative to this reference value.

### Assessment of cell wall polysaccharides content by fluorescence microscopy

The methodology for the staining of the cell wall polysaccharides was adapted from Pradhan et al.^43^. Concanavalin A-FITC, Fc-Dectin 1-Alexa 633 and Calcofluor White were used as staining compounds that bind mannans, glucans and chitin, respectively. Yeast cells were harvested at adequate time-points during cultivation as described above. Cells at an OD_600nm_ of 0.5 were incubated with 0.75 µg/mL Fc-Dectin 1 (Sino Biological, China) dissolved in FACS buffer for 45 min on ice. After centrifugation at 4300*g* for 5 min at 4 °C, the pellet was washed with 200 µL FACS buffer. Cells were then incubated for 30 min with 1:200 of anti-human IgG conjugated with Alexa Fluor 633 (ThermoFisher Scientific, Waltham, USA), 50 µg/mL Calcofluor White (Fluka) and 50 µg/mL Concanavalin A conjugated with Fluorescein (Sigma). After centrifugation at 4300*g* for 5 min at 4 °C, the pellet was washed with 200 µL FACS buffer. Stained cells were resuspended in 100 µL FACS buffer and were immobilized in 2.2% agarose in PBS 1× mounted on a gene frame 1.0 × 1.0 cm (ThermoFisher Scientific).

All measurements were performed using a Leica TCS SP5 (Leica Microsystems CMS GmbH, Mannheim, Germany) inverted confocal microscope (DMI6000). A 63× apochromatic water immersion objective with a NA of 1.2 (Zeiss, Jena Germany) was used for all experiments. Images were collected at 2048 × 2048 resolution. For the Concanavalin A-FITC channel, confocal microscopy measurements were carried out with an Argon laser for excitation at 488 nm. Emission was collected between 495 and 580 nm. For the FC-Dectin1-Alexa 633 channel, confocal microscopy was employed as well, and excitation was carried out through a He–Ne laser line at 633 nm. Fluorescence emission was collected in this channel between 640 and 770 nm. Finally, 2-photon excitation microscopy was used for the Calcofluor White channel with excitation from a Spectra-Physics Mai Tai BB laser set at 780 nm. Calcofluor white emission was collected between 390 and 480 nm. Data analysis was carried out using ImageJ. Regions of interest (ROI) corresponding to the cellular surface were defined and average pixel fluorescence intensities within these ROIs were determined for each channel and average background fluorescence was always subtracted to the calculated values.

The staining of the cells was carried out to ensure that the analysis of mannans, β-glucans and chitin refers to the same cell population. Results of the median intensity fluorescence were obtained from the analysis of at least 34 cells obtained from two independent experiments.

## Supplementary Information

Supplementary Information.

## Data Availability

All data generated or analysed during this study are included in this published article (and its Supplementary Information files).

## References

[1] Palma, M. & Sá-Correia, I. Physiological Genomics of the Highly Weak-Acid-Tolerant Food Spoilage Yeasts of *Zygosaccharomyces bail*ii sensu lato. in *Progress in molecular and subcellular biology *vol. 58 85–109 (Springer International Publishing, 2019). 10.1007/978-3-030-13035-0_4 30911890

[2] Palma M, Guerreiro JF, Sá-Correia I (2018). Adaptive response and tolerance to acetic acid in *Saccharomyces cerevisiae* and *Zygosaccharomyces bailii*: A physiological genomics perspective. Front. Microbiol..

[3] Cunha JT, Romaní A, Costa CE, Sá-Correia I, Domingues L (2019). Molecular and physiological basis of *Saccharomyces cerevisiae* tolerance to adverse lignocellulose-based process conditions. Appl. Microbiol. Biotechnol..

[4] Lide, D. R. DISSOCIATION CONSTANTS OF ORGANIC ACIDS AND BASES. in *CRC Handbook of Chemistry and Physics* 8–46 (CRC Press, 2003).

[5] Kawahata M, Masaki K, Fujii T, Iefuji H (2006). Yeast genes involved in response to lactic acid and acetic acid: Acidic conditions caused by the organic acids in *Saccharomyces cerevisiae* cultures induce expression of intracellular metal metabolism genes regulated by Aft1p. FEMS Yeast Res..

[6] Mira NP, Palma M, Guerreiro JF, Sá-Correia I (2010). Genome-wide identification of *Saccharomyces cerevisiae* genes required for tolerance to acetic acid. Microb. Cell Fact..

[7] Abbott DA (2007). Generic and specific transcriptional responses to different weak organic acids in anaerobic chemostat cultures of *Saccharomyces cerevisiae*. FEMS Yeast Res..

[8] Li BZ, Yuan YJ (2010). Transcriptome shifts in response to furfural and acetic acid in *Saccharomyces cerevisiae*. Appl. Microbiol. Biotechnol..

[9] Mira NP, Becker JD, Sá-Correia I (2010). Genomic expression program involving the Haa1p-regulon in *Saccharomyces cerevisiae* response to acetic acid. Omi. A J. Integr. Biol..

[10] Bajwa PK (2013). Transcriptional profiling of *Saccharomyces cerevisiae* T2 cells upon exposure to hardwood spent sulphite liquor: Comparison to acetic acid, furfural and hydroxymethylfurfural. Antonie van Leeuwenhoek Int. J. Gen. Mol. Microbiol..

[11] Dong Y, Hu J, Fan L, Chen Q (2017). RNA-Seq-based transcriptomic and metabolomic analysis reveal stress responses and programmed cell death induced by acetic acid in *Saccharomyces cerevisiae*. Sci. Rep..

[12] Antunes M, Palma M, Sá-correia I (2018). Transcriptional profiling of *Zygosaccharomyces bailii* early response to acetic acid or copper stress mediated by ZbHaa1. Sci. Rep..

[13] Longo V (2015). Proteome and metabolome profiling of wild-type and YCA1-knock-out yeast cells during acetic acid-induced programmed cell death. J. Proteomics.

[14] Almeida B (2009). Yeast protein expression profile during acetic acid-induced apoptosis indicates causal involvement of the TOR pathway. Proteomics.

[15] Godinho CP (2018). Pdr18 is involved in yeast response to acetic acid stress counteracting the decrease of plasma membrane ergosterol content and order. Sci. Rep..

[16] Lindberg L, Santos AXS, Riezman H, Olsson L, Bettiga M (2013). Lipidomic profiling of *Saccharomyces cerevisiae* and *Zygosaccharomyces bailii* reveals critical changes in lipid composition in response to acetic acid stress. PLoS ONE.

[17] Lindahl L, Genheden S, Eriksson LA, Olsson L, Bettiga M (2016). Sphingolipids contribute to acetic acid resistance in *Zygosaccharomyces bailii*. Biotechnol. Bioeng..

[18] Godinho CP, Sá-Correia I, Sá-Correia I (2019). Physiological genomics of multistress resistance in the yeast cell model and factory: Focus on MDR/MXR transporters. Yeasts in Biotechnology and Human Health–Physiological Genomic Approaches.

[19] Ullah A, Chandrasekaran G, Brul S, Smits GJ (2013). Yeast adaptation to weak acids prevents futile energy expenditure. Front. Microbiol..

[20] Francois JM (2016). Cell surface interference with plasma membrane and transport processes in yeasts. Adv. Exp. Med. Biol..

[21] Dague E (2010). An atomic force microscopy analysis of yeast mutants defective in cell wall architecture. Yeast.

[22] Schiavone M, Déjean S, Sieczkowsk N, Dague E, François JM (2017). Integration of biochemical, biophysical and transcriptomics data for investigating the structural and nanomechanical properties of the yeast cell wall. Front. Microbiol..

[23] Pillet F (2014). Uncovering by Atomic Force Microscopy of an original circular structure at the yeast cell surface in response to heat shock. BMC Biol..

[24] Schiavone M (2016). An Atomic Force Microscopy study of yeast response to ethanol stress: Evidence for a role of the plasma membrane in the nanomechanical properties of the cell walls. Appl. Environ. Microbiol..

[25] Kitichantaropas Y (2016). Cellular mechanisms contributing to multiple stress tolerance in *Saccharomyces cerevisiae* strains with potential use in high-temperature ethanol fermentation. AMB Express.

[26] Cunha JT (2018). *HAA1* and *PRS3* overexpression boosts yeast tolerance towards acetic acid improving xylose or glucose consumption: Unravelling the underlying mechanisms. Appl. Microbiol. Biotechnol..

[27] Kapteyn JC (2001). Low external ph induces HOG1-dependent changes in the organization of the *Saccharomyces cerevisiae* cell wall. Mol. Microbiol..

[28] Simões T, Mira NP, Fernandes AR, Sá-Correia I (2006). The *SPI1* gene, encoding a glycosylphosphatidylinositol-anchored cell wall protein, plays a prominent role in the development of yeast resistance to lipophilic weak-acid food preservatives. Appl. Environ. Microbiol..

[29] Jung US, Sobering AK, Romeo MJ, Levin DE (2002). Regulation of the yeast Rlm1 transcription factor by the Mpk1 cell wall integrity MAP kinase. Mol. Microbiol..

[30] Douglas CM (2001). Fungal β(1,3)-d-glucan synthesis. Med. Mycol. Suppl..

[31] Aimanianda V (2017). The dual activity responsible for the elongation and branching of β-(1,3)- glucan in the fungal cell wall. MBio.

[32] Gohlke S, Muthukrishnan S, Merzendorfer H (2017). In vitro and in vivo studies on the structural organization of Chs3 from *Saccharomyces cerevisiae*. Int. J. Mol. Sci..

[33] Blanco N (2015). Structural and functional analysis of yeast Crh1 and Crh2 transglycosylases. FEBS J..

[34] Ram AFJ (1998). Loss of the plasma membrane-bound protein Gas1p in *Saccharomyces cerevisiae* results in the release of β1,3-glucan into the medium and induces a compensation mechanism to ensure cell wall integrity. J. Bacteriol..

[35] Mira NP, Teixeira MC, Sá-Correia I (2010). Adaptive response and tolerance to weak acids in *Saccharomyces cerevisiae*: A genome-wide view. OMICS.

[36] Guerreiro JF, Muir A, Ramachandran S, Thorner J, Sá Correia I (2016). Sphingolipid biosynthesis upregulation by TOR complex 2-Ypk1 signaling during yeast adaptive response to acetic acid stress. Biochem. J..

[37] Mollapour M, Shepherd A, Piper PW (2009). Presence of the Fps1p aquaglyceroporin channel is essential for Hog1p activation, but suppresses Slt2(Mpk1)p activation, with acetic acid stress of yeast. Microbiology.

[38] Ando A, Nakamura T, Murata Y, Takagi H, Shima J (2007). Identification and classification of genes required for tolerance to freeze-thaw stress revealed by genome-wide screening of *Saccharomyces cerevisiae* deletion strains. FEMS Yeast Res..

[39] Lucena RM, Dolz-Edo L, Brul S, de Morais MA, Smits G (2020). Extreme low cytosolic pH is a signal for cell survival in acid stressed yeast. Genes.

[40] Udom N, Chansongkrow P, Charoensawan V, Auesukaree C (2019). Coordination of the cell wall integrity and highosmolarity glycerol pathways in response to ethanol stress in *Saccharomyces cerevisiae*. Appl. Environ. Microbiol..

[41] Sader JE (2016). A virtual instrument to standardise the calibration of atomic force microscope cantilevers. Rev. Sci. Instrum..

[42] Collart. M. A. & Oliviero, S. Preparation of yeast RNA. *Curr. Protoc. Mol. Biol*. **13**(13), 12 (2001). 10.1002/0471142727.mb1312s23 18265096

[43] Pradhan A (2018). Hypoxia promotes immune evasion by triggering-glucan masking on the *Candida albicans* cell surface via mitochondrial and cAMP-protein kinase A signaling. MBio.

