# Supplementary material for: Yeast adaptive response to acetic acid stress involves structural alterations and increased stiffness of the cell wall
Source: Sci Rep. 2021 Jun 16;11:12652. doi: 10.1038/s41598-021-92069-3 (PMC8209030; doi:10.1038/s41598-021-92069-3)
Supplement: Supplementary file 1 — Supplementary Information. [file 41598_2021_92069_MOESM1_ESM.pdf]

Yeast adaptive response to acetic acid stress involves structural alterations and increased stiffness of the cell wall

Ricardo A. Ribeiro, Miguel V. Vitorino[+], Cláudia P. Godinho[+], Nuno Bourbon-Melo[+], Tiago T. Robalo, Fábio Fernandes, Mário S. Rodrigues, and Isabel Sá-Correia[*]

[+] these three authors should be regarded as joint second authors

[*] Correspondence: Prof. Dr. Isabel Sá-Correia
isacorreia@tecnico.ulisboa.pt

Supplementary Table S1 - Primers used for qRT-PCR analysis.

Primer	Sequence (5'-3')
ACT1	fw: CTCCACCACTGCTGAAAGAGAA<br><br>rev: CCAAGGCGACGTAACATAGTTTT
CHS3	fw: TCACCTGGATGTTTTACCATCAAG<br><br>rev: CCACTCCGACGAGTTGCAT
FKS1	fw: CATGCTGCTCTGGTCCCTTATT<br><br>rev:CACCGTGGGCAATTCCA
FKS2	fw: GCTCATGTCGTTGGAGCAGTT<br><br>rev: CCAATGGCATTACGGAAAAGA
RLM1	fw: CTTTTTTCTGCAACACAGCCATA<br><br>rev: CGCCAGGAATATTCGATGGT
GAS1	fw: AACCGCTGCTGCTTTTTTTG<br><br>rev: CTTCAATCGCTGGAACATCGT
CRH1	fw: CGCGGCTGCCGAAAG<br><br>rev: GCA GTGCTAGAAGCTGCAGTTG
BGL2	fw: TTTTGTTATGGCTAACGCGTTCT<br><br>rev: GAGTAAGAGGCATTTTGCATGGT
PRM5	fw: TTTTCCACACAACATACCCAGTTT<br><br>rev: TCTTTGGCGGGATAATCCATA

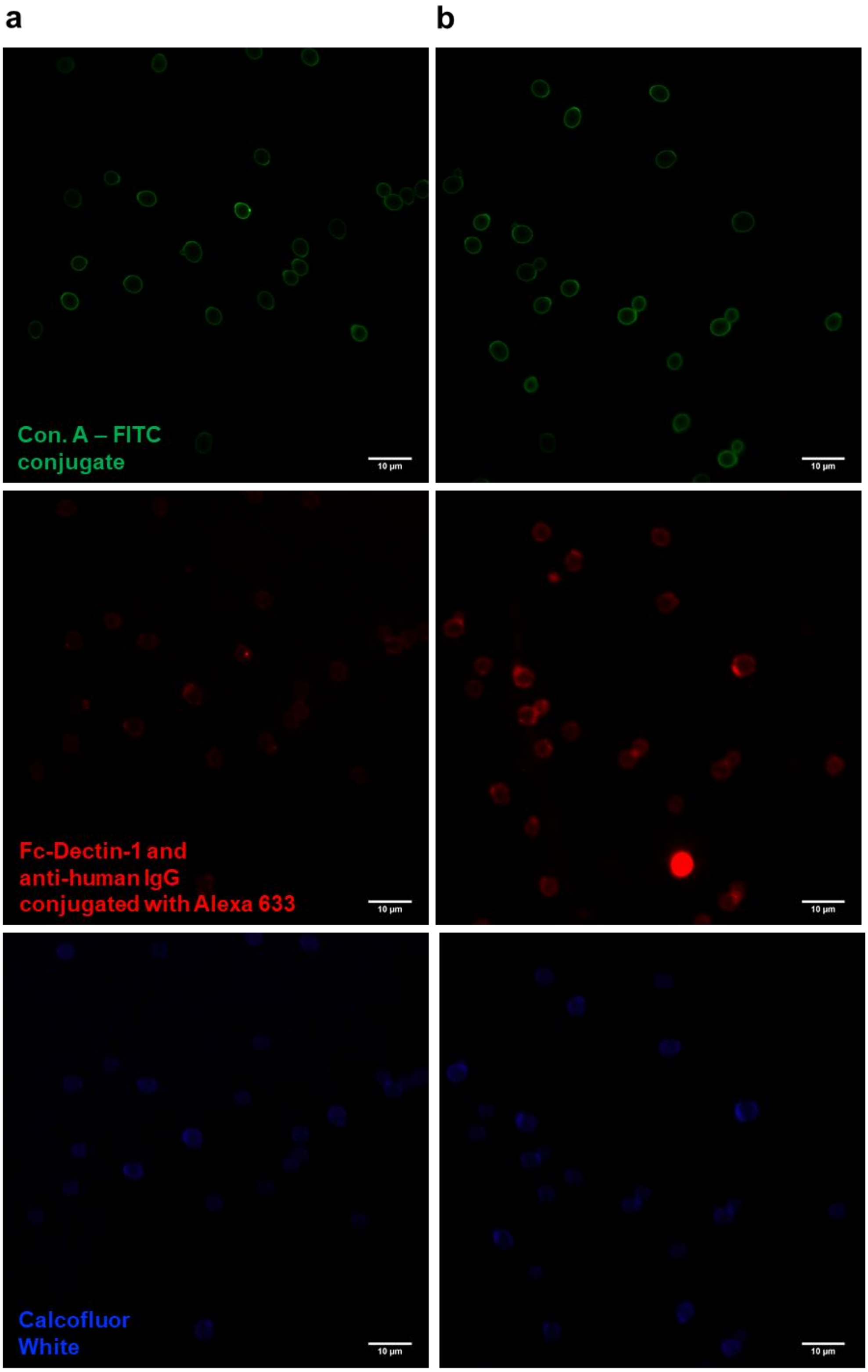

Supplementary Figure S1 - Fluorescence microscopy images of fluorescence-stained cell wall polysaccharides. Illustrative images of stained BY4741 cells were taken at 0 hours (a) and 12 hours (b) of exposure to acetic acid, corresponding to the end of the acetic acid-induced latency. Images were used to quantify cell wall mannans and β-glucans, using confocal microscopy, and chitin, using 2-photon excitation microscopy.

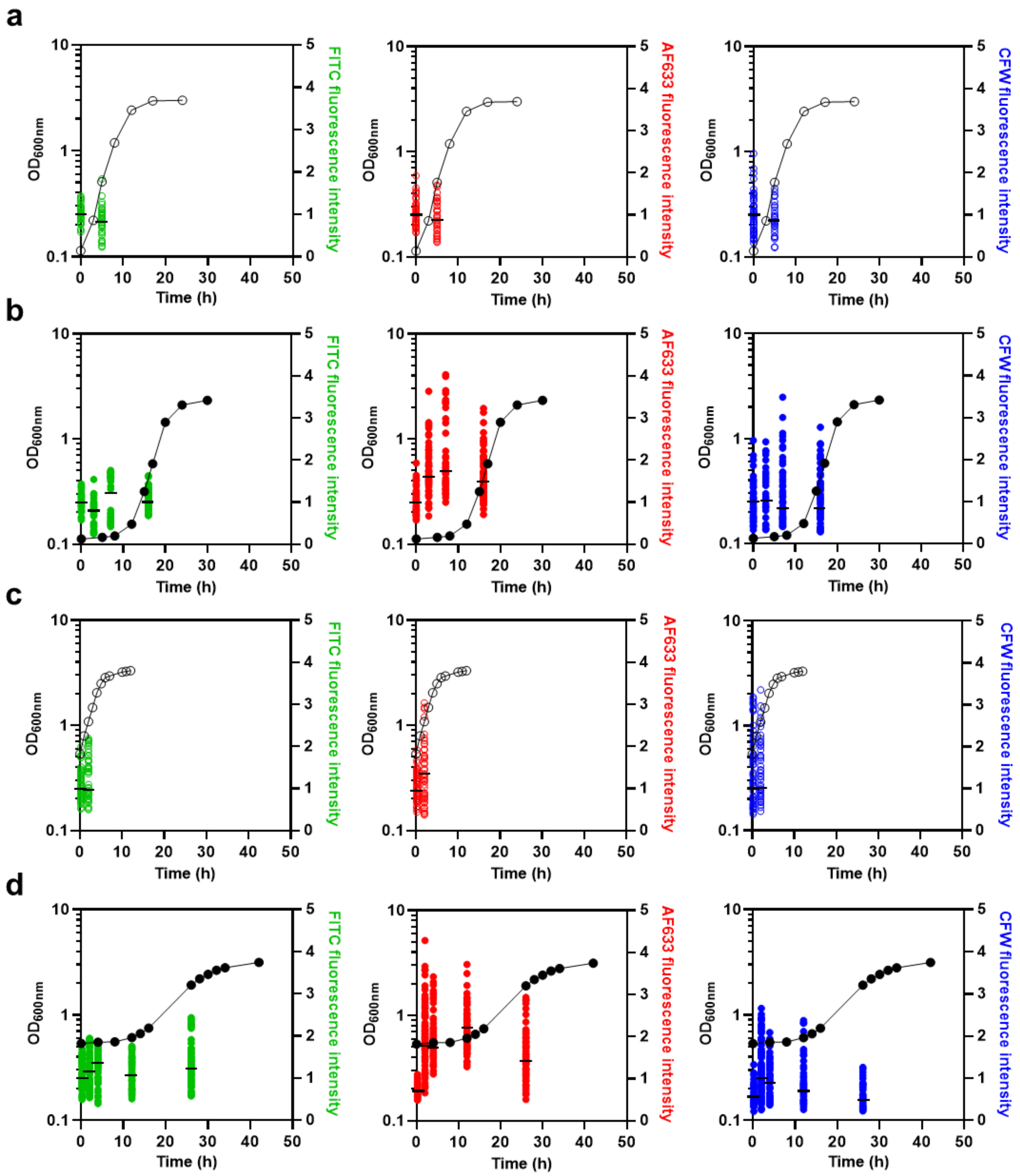

Supplementary Figure S2 – Content of cell wall polysaccharides in yeast cells during adaptation to acetic acid stress by fluorescence microscopy. Quantitative analysis of the cell wall polysaccharides was performed during cultivation of BY4741 cells in the absence (○; **a** and **c**) or presence (●; **b** and **d**) of 60 mM acetic acid (pH 4.0) at an initial OD 600nm of 0.1 (**a-b**) or 0.5 (**c-d**). The cell wall components mannans (green), β-glucans (red) and chitin (blue) were stained with Concanavalin A conjugated with Fluorescein (FITC), Fc-Dectin 1 conjugated with Alexa Fluor 633 (AF633), and Calcofluor White (CFW), respectively. Quantification of the fluorescence intensity was performed with a Leica TCS SP5 (Leica Microsystems CMS GmbH, Manheim, Germany) inverted confocal microscope (DMI600). Median fluorescence intensity is indicated by a dash and results from the analysis of, at least, 34 cells obtained from two independent experiments.
